# The effect of leisure engagement on preschool teachers’ job stress and sustainable well-being

**DOI:** 10.3389/fpsyg.2022.912275

**Published:** 2022-07-22

**Authors:** Liying Nong, Yu-Feng Wu, Jian-Hong Ye, Chen Liao, Changwu Wei

**Affiliations:** ^1^School of Education and Music, Hezhou University, Hezhou, China; ^2^Office of Physical Education, Ming Chi University of Technology, New Taipei City, Taiwan; ^3^Faculty of Education, Beijing Normal University, Beijing, China; ^4^College of Tourism and Sport Health, Hezhou University, Hezhou, China

**Keywords:** preschool teachers, leisure engagement, job stress, sustainable well-being, behavioral–emotional–cognitive

## Abstract

The preschool education profession often faces many challenges and preschool teachers, as important members of the preschool education profession must respond to a variety of emergencies with young children, which also leads to high levels of job stress and can have a negative impact on their ongoing well-being. Past research has pointed out that a healthy lifestyle is one of the key factors in enhancing sustainable well-being in high-stress work situations and many studies have found that good leisure activity engagement as a healthy lifestyle is associated with higher levels of well-being. However, the relationship between preschool teachers’ leisure engagement and sustainable well-being has been less explored. Therefore, this study proposed seven research hypotheses based on the engagement theory proposed by Fredricks et al. (2004) and developed a research model to explore the relationship between three types of leisure engagement, including behavioral, affective and cognitive, and preschool teachers’ job stress and sustainable well-being, using age as a control variable. This study used a cross-sectional web-based questionnaire with a convenience sample of 500 preschool teachers in China. The collected data were analyzed for reliability and validity, model fit testing and structural equation modeling for model validation after removing invalid data and incomplete responses. The results of the study showed that (a) behavioral engagement was not related with either the job stress or sustainable well-being of preschool teachers; (b) Emotional and cognitive engagement were negatively related to job stress but positively related to the sustainable well-being of preschool teachers; and (c) Job stress was negatively related to the sustainable well-being of preschool teachers; (d) Age is an effective control variable. From the above results, it is clear that not all three types of leisure engagement are effective in terms of reducing the work stress of preschool teachers. As well as being related to the sustainable well-being of preschool teachers emotional and cognitive engagement contributed more to sustainable well-being acquisition.

## Introduction

In 2015 the United Nations (UN) established 17 Sustainable Development Goals (SDGs) for the 2030 Agenda for Sustainable Development, aiming to eradicate poverty, implement quality education, ensure prosperity and health for all (United Nations, 2015). Among these, SDG 3 emphasizes “ensuring healthy lives and promoting well-being for all people of all ages”, with a greater focus on people’s mental health, healthy lifestyles and well-being (Sweileh, 2020). Moreover, while well-being is seen as a key sustainable goal (Di Fabio, 2017), sustainable well-being is also seen as a positive and continuous state of achieving inner peace and satisfaction in the face of various challenges and changes (Deng et al., 2020) and also as a state of putting continuous effort into work and life to continuously maintain a stable, satisfying and positive emotional experience (Sheldon and Lyubomirsky, 2021). This not only helps to promote teachers’ self-empowerment, but also motivates them to continue to be positive and healthy in their future work and life, thus promoting the acquisition of high levels of sustainable well-being (Hendrickson and Francis, 2018).

However, the global COVID-19 pandemic has hindered this goal and many people are under great stress and burdens due to the ongoing impact of the epidemic, which affects their mental health and well-being (Pellerin and Raufaste, 2020). Especially in the field of kindergarten education organization, preschool teachers, as important members of the preschool education industry have to face a variety of challenges from the preschool education industry in addition to dealing with various emergencies brought about by the impact of the epidemic, which may also lead to high levels of work stress and burnout (Duran, 2021). In addition, preschool teachers generally face long working hours, heavy workloads, low social status and low pay. If this state continues, it may not only lead to a series of negative effects such as burnout and turnover, but may also affect their expectations for their future work and life and is not conducive to the acquisition of the sustainable well-being of preschool teachers (Eadie et al., 2021). In contrast, sustainable well-being is an important factor in maintaining motivation at work and in life in general. Therefore, sustainable well-being is critical to the psychological well-being of preschool teachers and to their future development.

Health psychology aims to show how behaviors and lifestyles affect the psychological state of individuals (Schulz et al., 2018). Past research has focused more on the effects of individual factors such as self-efficacy, self-compassion and positive thinking on teachers’ work stress and well-being (Jennings, 2015; Zee and Koomen, 2016; Hwang et al., 2019), but the issue of how they sustain well-being at work and in life through an externally healthy lifestyle is also important. This is because a healthy lifestyle that includes leisure activities not only relieves their stressful state but is also a key factor in enhancing well-being (Hayosh, 2017; Hartman et al., 2020). Among these, leisure engagement, as a healthy lifestyle is commonly defined as the frequency with which individuals engage in leisure activities and is the primary driver that empowers individuals to continue to maintain a sense of passion, pleasure and purpose in their current and future lives (Fancourt et al., 2021).

Research also confirms the strong relationship between sustained well-being and leisure engagement (Wheatley and Bickerton, 2019). In addition, preschool teachers are important members of the kindergarten education profession and are exposed to high levels of work stress over time (Swigonski et al., 2021). Leisure activities may be an important factor in relieving their physical and mental stress as well as maintaining their levels of well-being. In other words, leisure engagement is critical for preschool teachers to continue to be enthusiastic about their work and for them to maintain a sense of well-being in the face of highly varied and challenging work situations. For example, participation in leisure activities can not only gradually strengthen their leisure behavior, but can also change their cognitive and psychological state, so that they can continue to be satisfied and grow in their work and life in the future (Tsaur et al., 2021). This state of leisure engagement is not only a behavioral engagement, but also cognitive and emotional engagement is equally important for continued satisfaction, pleasure and well-being in subsequent work and life (Matsumoto et al., 2018). Therefore, the purpose of this study was to explore the relationship between leisure engagement and the sustained well-being of preschool teachers.

In addition, according to the World Health Organization, stress has been described as the “health epidemic of the 21st century” (Tang and Duan, 2021). In organizational work, job stress is a negative physiological or psychological response to intense work demands (Burman and Goswami, 2018; Xie et al., 2021) and each individual exhibits a different job stress situation. In addition, a previous study also found that preschool teachers were subjected to high levels of job stress due to various challenges and difficulties in their work situations, resulting in low levels of well-being (Li and Zhang, 2019) and that this may further affect the continued well-being of preschool teachers in their future work and life. In addition, related research has shown that leisure engagement facilitates a protective barrier that relieves high levels of stress through relaxation, recreation and other leisure activities, prompting individuals to protect themselves from the negative effects of work and life (Hayosh, 2017). Therefore, leisure engagement may affect the sustainable well-being of preschool teachers through the stress pathway of job stress.

According to the engagement theory proposed by Fredricks et al. (2004), engagement should be a multidimensional structure that includes cognitive, emotional, as well as behavioral engagement. In contrast, the behavioral, cognitive, and affective components of engagement as an important element of the motivational process are interconnected with the individual’s environment and activities, are dynamically embedded in individual systems and outcomes, and provide a rich resource for individual growth and development (Wang and Fredricks, 2014). Therefore, using Fredricks et al.’s (2004) engagement theory as a theoretical basis for sustainable well-being would serve to strengthen the research discourse.

Among them, leisure behavioral engagement is a manifestation and frequency of active participation in leisure activities (Odom, 2016) and more leisure behavioral engagement, such as participation in sports activities, singing karaoke, and walking, would be beneficial for relieving preschool teachers’ work stress and tension (Petrić et al., 2022). In addition, affective engagement is a feeling that people have about participating in leisure activities, which refers to the degree of good or bad feelings toward leisure activities and leisure experiences; moreover, it is a direct feeling of interest and expectation regarding leisure activities and their experiences (Wu et al., 2021), including the emotional disposition that preschool teachers show toward participating in leisure activities. Cognitive engagement, on the other hand, is a form of self-strategic regulatory learning (Wang and Fredricks, 2014) and also refers to preschool teachers’ intellectual understanding of leisure activities and leisure experiences. A previous study showed a positive relationship between leisure engagement and well-being. Previous research has shown that leisure engagement relieves work stress and is positively associated with well-being (Chiu et al., 2020). More importantly, in a challenging and difficult work situation, preschool teachers may be able to alleviate to some extent of their high levels of work stress and be influenced to maintain a state of sustained well-being by engaging in more leisure activities and by being aware of the importance of health and actively participating in leisure activities. Therefore, exploring the three types of leisure engagement is critical to understanding how preschool teachers maintain a sense of sustainable well-being.

From the above, it can be inferred that by engaging in leisure activities, preschool teachers will help maintain a healthy lifestyle to reduce work stress and influence their ability to maintain a sustained sense of well-being in their future work and life. However, current research on the relationship between leisure and well-being has focused more on exploring leisure activities for groups such as adolescents and the elderly, and there is still a gap in exploring the relationship between the three types of leisure engagement and sustainable well-being among preschool teachers. Therefore, in order to further understand the relationship between preschool teachers’ leisure engagement and sustainable well-being, this study built on the engagement theory proposed by Fredricks et al. (2004) and developed a research model to explore the relationship between the three types of leisure engagement, namely behavioral, affective, and cognitive engagement, and preschool teachers’ job stress and sustainable well-being.

### The relationship between behavioral engagement and job stress

Research has shown that leisure activity is a significant predictor of people’s health (Kim and Brown, 2018). Leisure behavioral engagement is commonly defined as the frequency of participation in leisure activities and behavioral performance (Nagata et al., 2020) and may be an important way to reduce work stress among preschool teachers. A related study found that many preschool teachers experienced a sharp increase in stress and tension due to the impact of the epidemic (Hong et al., 2021), especially with the closure of gyms and other recreational areas, which not only limited their leisure behavioral engagement, resulting in high levels of stress that could not be released, but also had a negative impact on their physical and mental health (Kim et al., 2020). Those who regularly engage in leisure activities such as physical activity, travel, volunteering, and recreation can reduce their mental health problems such as depression and anxiety through leisure activities (Morse et al., 2021). In addition, those teachers who regularly engage in leisure activities have lower levels of job stress in challenging work environments (Chen, 2016). Therefore, the hypothesis of this study based on the kindergarten work context on the leisure behavior engagement and work stress of preschool teachers is that behavioral engagement is negatively related to job stress (H1).

### The relationship between behavioral engagement and sustainable well-being

According to the sustainable well-being model, when people are committed to the pursuit of happiness and are actively and consistently engaged in these activities in various forms, such a state of engagement may in turn provide them with a stable, positive emotional experience while also increasing their source motivation to continue to feel happy (Sheldon and Lyubomirsky, 2021). Due to the challenges and difficulties often faced in kindergarten work situations, preschool teachers have higher levels of job stress and tendency to leave compared to other types of teachers (Eadie et al., 2021). Teachers who have higher levels of leisure behavioral engagement will have higher levels of well-being (Cho, 2021), which also affects their ability to continue engaging in positive, sustainable experiences of well-being in the future (Hendrickson and Francis, 2018). Therefore, leisure activities are crucial to the well-being and mental health of preschool teachers. In contrast, people who regularly engage in leisure activities are more likely to continue to have positive emotional experiences as their physical condition improves and their stress is relieved in the face of challenging environments and work (Doistua et al., 2020; Tian et al., 2020). In other words, more leisure behavioral engagement by preschool teachers implies higher levels of sustainable well-being. Therefore, the hypothesis of this study on leisure behavior engagement and sustainable well-being of preschool teachers is that behavioral engagement is positively related with sustainable well-being (H2).

### The relationship between emotional engagement and job stress

Stress often occurs when environmental demands exceed an individual’s resources and are difficult to meet (Duan and Mu, 2018) and the continuation of such high levels of stress may jeopardize people’s mental health and expectations for a better life (Huang et al., 2021). Especially in the field of kindergarten education, preschool teachers have more work stress than teachers at other stages of education and the relatively low cognitive level of young children and the variety of work demands they often face not only negatively affect preschool teachers’ positive emotions, but also lead to high levels of work stress (Hong et al., 2022; Nong et al., 2022). However, leisure activity users with positive emotions may alleviate such stressful states by engaging in more leisure activities (Chen et al., 2020). In addition, research has indicated that those with higher leisure emotional engagement tend to have lower stress levels and providing more leisure emotional engagement and increased autonomy in leisure activities may be an important means of effectively reducing people’s work stress (Chang, 2018). This implies that preschool teachers’ emotional attitudes toward leisure activities may influence their job stress status. Therefore, the hypothesis of this study on leisure emotional engagement and job stress of preschool teachers is that emotional engagement is negatively related to job stress (H3).

### The relationship between emotional engagement and sustainable well-being

Research suggests that people’s ability to maintain a state of well-being depends on whether they have a peaceful state of mind and positive emotional experiences in the face of difficulties and setbacks (Määttä and Uusiautti, 2020). Sustainable well-being, on the other hand, refers to people’s ability to sustain stable, positive emotional experiences in their work and life (Sheldon and Lyubomirsky, 2021). In addition, researchers have indicated that sustained positive affective experiences bring higher levels of well-being to preschool teachers (Lee et al., 2022). For example, teachers of young children who enjoy participating in leisure activities have more opportunities for social interaction, which also provides them with more positive interactions and facilitates the acquisition of positive emotional affect. In addition, regular participation in leisure activities promotes positive psychological states, which in turn buffer some job stress by enhancing positive emotions, further contributing to a sustained increase in emotional well-being (Chen et al., 2020). In particular, it is important to note that a variety of work demands and environmental changes can have a significant impact on preschool teachers’ job status (Swigonski et al., 2021) and that emotional engagement through leisure can help them recover more quickly from negative life events, engage in leisure activities with positive emotions, better balance work and life, and consistently impact mental health and well-being (Cho, 2021). Therefore, the hypothesis of this study on the relationship between emotional engagement and sustainable well-being of preschool teachers is that emotional engagement is positively related with sustainable well-being (H4).

### The relationship between cognitive engagement and job stress

Leisure cognition refers to people’s knowledge and beliefs about their participation in leisure activities, specifically the belief that leisure activities contribute to the development of one’s physical and mental health, as well as to one’s expertise, sources of leisure information and self-development (Wu et al., 2021). Related studies suggest that greater cognitive engagement in leisure is strongly associated with better mental health, with important effects on reducing work stress, regulating negative emotions and burnout (Iwasaki et al., 2001; Rodríguez-Rey et al., 2020). Those with a higher cognitive investment in leisure tend to value leisure activities and will invest more time and energy in leisure activities, which may be beneficial for relieving the tension of being under a chronically high load (Poulin et al., 2019). Due to the dual responsibility of child care and education, as well as the various tasks and unexpected situations that often arise in their daily work, preschool teachers are generally perceived to be in a state of high stress and strain over time (Hong et al., 2022). If preschool teachers are aware of the importance of participating in leisure activities, they will be more likely to reduce their work stress and maintain their energy and enthusiasm for their future work and life by engaging in more leisure activities (Petrić et al., 2022). In other words, higher cognitive engagement in leisure among preschool teachers implies a lower level of stressful states through which leisure activities contribute to improving their health and provide some resources for coping with various stressors in complex environments. Therefore, the hypothesis of this study on cognitive engagement and job stress of preschool teachers is that cognitive engagement is negatively related to job stress (H5).

### The relationship between cognitive engagement and sustainable well-being

Leisure activities as a healthy lifestyle have a positive impact on people’s health and quality of life (Belo et al., 2020). In addition, people who engage in leisure activities continuously maintain normal cognitive and physical functioning, and their physical and mental stress can be relieved from leisure activities which continue to influence their mental health (Sala et al., 2019). For example, those who are more engaged in leisure activities have more leisure knowledge and experience, develop more adaptive skills, social interactions and positive relationships through leisure activities (Doistua et al., 2020). Moreover, a recent study also indicated that preschool teachers should value the importance of leisure activities such as play and recreation and engage in more leisure activities to enhance their physical and mental health as well as that of preschool children (Cheung, 2020). In addition, recognizing the important role of leisure activities is more beneficial for helping them understand the relationship between leisure and health, well-being and work, thus continuously influencing their positive emotions, strengthening their social connections and promoting the acquisition of sustainable well-being (Bailey et al., 2016). At the same time, a recent study confirmed the close relationship between leisure cognitive engagement and well-being (Matsumoto et al., 2022). This means that the higher the cognitive engagement in leisure, the higher the sustainable well-being of preschool teachers is likely to be. Therefore, the hypothesis of this study on cognitive engagement and sustainable well-being of preschool teachers is that cognitive engagement is positively related with sustainable well-being (H6).

### The relationship between job stress and sustainable well-being

Recent studies have indicated that job stress is an important factor that hinders people’s well-being acquisition (Tandler et al., 2020). In addition, studies have also found that kindergarten teachers generally suffer from high job stress, burnout and emotional management problems (Jeon et al., 2018). For example, preschool teachers often face a range of stresses and challenges, such as heavy workloads, severe lack of leisure time and workloads that are not proportional to their pay, which may severely damage their psychological health and negatively affect their well-being (Li and Zhang, 2019). In addition, related studies have also confirmed that excessive job stress may produce anxiety and tension in preschool teachers, which may also lead to lower job satisfaction and consistently affect well-being attainment (Hong et al., 2021). In other words, lower job stress is related with higher levels of sustainable well-being. More importantly, in the current complex environment and challenges, it is important to give preschool teachers more support to cope with the stress in the work environment with a positive mindset through reducing psychological burdens (Jeon et al., 2018), so that they can maintain a continuous sense of well-being in their future work and life and bring more contributions to the kindergarten organization (Lee et al., 2022). Therefore, this study explores the relationship between job stress and sustainable well-being of preschool teachers and proposes the hypothesis that job stress is negatively related with sustainable well-being (H7).

### Effect of age

Studies have found that people’s leisure engagement and happiness levels are related to many factors, such as stress, psychological needs, leisure time, and age (Babenko et al., 2018; Hartman et al., 2020). In addition, demographic factors such as age are also an important factor influencing people’s leisure activities and well-being (Cocozza et al., 2020). Moreover, in a study of adults aged 25 to 66, age was found to be strongly associated with leisure engagement and well-being, with those of lower age tending to have lower leisure engagement and also lower levels of those with lower ages tend to have lower leisure engagement and lower levels of well-being (Chen et al., 2020). In addition, controlling variables can better reduce the effect of error on study results and can improve statistical efficacy (Becker, 2005). Therefore, in order to reduce the effect of control variables on the study results, this study used age as a control variable to further exclude the effect on the study results according to the above literature.

## Materials and methods

### Research model

**FIGURE 1:**
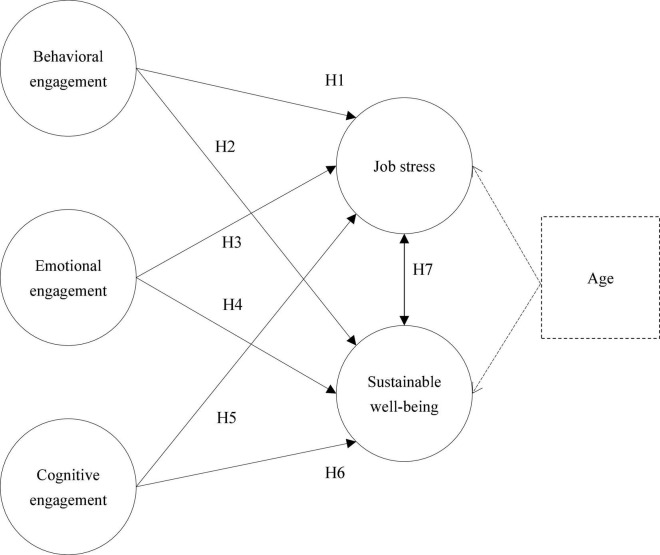
Research model. Note: Age as a control variable.

The engagement theory proposed by Fredricks et al. (2004) suggests that engagement is a multidimensional structure that contains behavioral, emotional, and cognitive components that facilitate the maintenance of positive and sustained individual motivation by being dynamically embedded in the individual’s environment and system, influencing individual growth and development (Wang and Fredricks, 2014). Therefore, to further understand the relationship between preschool teachers’ leisure engagement and sustainable well-being, Based on the engagement theory proposed by Fredricks et al. (2004), this study proposed seven research hypotheses to explore the relationship between three types of leisure engagement, such as behavioral, emotional, and cognitive, and early preschool teachers’ job stress and sustainable well-being, with age as a control variable by excluding age-related effects and constructed a research model (see Figure 1).

### Research procedure

This study used a cross-sectional web-based questionnaire that was completed by a purposive sample of 500 preschool teachers in China through the Wènjuànxīng (WJX) platform. The Wènjuànxīng (WJX) platform is an online survey platform similar to Google Form, through which researchers can post questionnaires online and respondents can complete them online. First, the researcher stated the purpose of the study, the use of the data, the privacy of the participants, and asked for informed consent on the first page of the questionnaire. Those who agreed to participate in the questionnaire were considered to have voluntarily participated in the survey. Therefore, the questionnaire for this study was completed only with the informed consent of the preschool teachers. Subsequently, the questionnaire information for this study was collected from January 15 to February 28, 2022 and the link to the questionnaire was closed after 500 questionnaires were collected.

### Participants

The number of participants in this study was 500 and a total of 51 invalid questionnaires with incomplete answers, overly fast completion time, and outlier values were deleted. The number of valid participants in this study was therefore 449, with a valid recovery rate of 89.8%. The background of the relevant participants is shown in Table 1.

**TABLE 1:** Demographic details of participants.

Variables	Content
Gender	Female: 437 (97.3%)
	Male: 12 (2.7%)
Age	18 to 30: 344 (76.6%)
	31 to 40: 75 (16.7%)
	41 to 50: 27 (6%)
	51 years and older: 3 (0.7%)
Overtime	Frequent overtime: 249 (55.5%)
	Infrequent overtime: 200 (44.5%)
Years of experience	Less than 1 year: 147 (32.7%)
	2 to 5 years: 206 (45.9%)
	6 to 10 years: 64 (14.3%)
	11 to 15 years: 18 (4%)
	16 to 20 years: 2 (0.4%)
	21 years and over: 12 (2.7%)
Type of kindergarten	Public: 280 (62.4%) Private: 169 (37.6%)
Number of days of participation in leisure activities per week	Little or no leisure time: 122 (27.1%)
	1–2 days: 302 (67.3%)
	3–4 days: 16 (3.6%)
	5–6 days: 4 (0.9%)
	Every day: 5 (1.1%)
Popular leisure activities	Static outdoor activities: 39 (8.7%)
	Moving outdoor activities: 23 (5.1%)
	Static indoor activities: 144 (32.1%)
	Moving indoor activities: 2 (0.4%)
	Social activities: 101 (22.5%)
	Entertainment activities: 84 (18.7%)
	Other: 56 (12.5%)

### Measurement

This study used a quantitative verification research model in which the instruments measured in the survey were primarily developed from past research and related theories through three rounds of validity reviews conducted by inviting three experts in the field of sports and leisure. Afterward, five preschool teachers were invited to try out the instrument and fill in the responses to ensure the face validity of the questionnaire. In addition, as the Likert 5-point scale has high reliability, a Likert 5-point scale was used as the standard for questionnaire content scale design in this study, where 1 represents *strongly disagree*, 2 represents *disagree*, 3 represents *neutral*, 4 represents *agree* and 5 represents *strongly agree*.

#### Leisure engagement

Leisure engagement as a healthy lifestyle refers to the state of behavioral, emotional, and cognitive engagement that people exhibit when they individually engage in leisure activities (Wu et al., 2021). Based on this definition, this study assessed the leisure engagement status of preschool teachers by developing 18 questions based on Fredricks’ (2004) three types of engagement. Examples of behavioral engagement are: “I usually participate in leisure activities on time and as planned” and “I actively participate in leisure activities.” Examples of emotional engagement include: “I like to spend time on leisure activities apart from work” and “I like to discuss leisure-related activities with colleagues.” Examples of cognitive engagement are: “I plan ahead before I do a leisure activity” and “I write down any important details of the leisure activity so I don’t forget them.”

#### Job stress

Job stress, as an assessment of people’s work status, is often seen as a physiological or psychological response to the inability to meet job requirements (Burman and Goswami, 2018). Based on the above definition, this study adapted Fimian’s (1984) eight-item teacher work stress scale to assess the job stress experienced by preschool teachers. Example items are: “I feel stressed because I have no control over what happens at work in relation to kindergarten” and “I feel stressed because I have to constantly monitor my behavior at work.”

#### Sustainable well-being

Sustainable well-being is defined as a stable, positive emotional experience that people continue to have at work and in their everyday life (Sheldon and Lyubomirsky, 2021). Based on this definition, the questionnaire was adapted from Lu and Lin’s (2003) very short version of the Chinese Well-Being Inventory, which consists of 10 items, to assess preschool teachers’ perceptions of sustainable well-being. Example items are: “I am continuously happy at work” and “I am optimistic about my future work life.”

## Results and discussion

Structural equation modeling, an important and widely applied method of statistical analysis for empirical research in the social sciences is commonly used to explore the structural relationships between validated latent variables because it can model the measured variables and account for various measurement errors (Astrachan et al., 2014). Therefore, this study first used SPSS to conduct item analysis and reliability analysis of the research instrument, then validation factor analysis of each research scale through AMOS statistical software, followed by structural equation modeling (SEM) to conduct model verification and path analysis for the proposed research model. Finally, in order to test the mediation effect of the study model, the non-parametric percentile Bootstrap method was also used to test the mediation effect of the study model (MacKinnon et al., 2004).

### Item analysis

Confirmatory factor analysis (CFA) is often used to measure the rationality of constructs, while first-order CFA is better at measuring the suitability of the items for each construct (Kline, 2015). First, the factor loading (FL) of each item was analyzed and dimension items with a FL value below 0.500 were deleted (Hair et al., 2010). Second, Hair et al. (2019) proposed that χ^2^/*df* should be less than 5, GFI greater than 0.80 and RMSEA less than 0.1. Therefore, first-order validated factor analysis CFA was used to confirm the internal validity of each item and the measured values were within the standard range (see Table 2). As a result, the number of items related to behavioral engagement, emotional engagement and cognitive engagement all decreased from six to five; job stress decreased from eight to six; and sustainable well-being decreased from 10 to eight, as shown in Figure 1.

**TABLE 2:** First-order confirmatory factor analysis (CFA).

Construct	χ^2^	*df*	χ^2^/df	RMSEA	GFI	AGFI	FL
Threshold	−	−	<5	<0.10	>0.80	>0.80	>0.5
Behavioral engagement	24.4	5	4.88	0.93	0.98	0.94	0.61–0.85
Emotional engagement	9.28	5	1.86	0.44	0.99	0.98	0.64–0.82
Cognitive engagement	17.68	5	3.54	0.75	0.99	0.96	0.73–0.89
Job stress	35.60	9	4.00	0.81	0.97	0.94	0.67–0.90
Sustainable well-being	66.75	14	4.77	0.92	0.96	0.92	0.80–0.89

### Reliability and validity analysis

Cronbach’s α and Composite Reliability (CR) were used to measure the reliability of the constructs. First, when the Cronbach’s alpha value and the CR value are 0.7 and above, the reliability of the construct is good (Cook and Beckman, 2006). Therefore, the SPSS statistical software was used to conduct the construct internal consistency reliability (Cronbach’s α) analysis in this study, as shown in Table 3, the Cronbach’s α values for each construct ranged from 0.86 to 0.94, indicating good reliability. In addition, the CR values measured in this study ranged from 0.88 to 0.95, all of which were higher than 0.7, which meets the criteria (Cook and Beckman, 2006).

**TABLE 3:** Reliability and validity analysis.

Construct	*M*	SD	α	FL	CR	AVE	*t*
Behavioral engagement	3.20	0.70	0.86	0.77	0.88	0.60	13.33–19.91
Emotional engagement	3.40	0.67	0.88	0.78	0.88	0.60	13.94–14.24
Cognitive engagement	3.32	0.63	0.88	0.80	0.90	0.64	15.57–19.33
Job stress	3.37	0.87	0.92	0.83	0.93	0.69	15.09–16.77
Sustainable well-being	3.32	0.77	0.94	0.85	0.95	0.73	19.92–22.72

Secondly, FL values above 0.50 for each item should indicate good convergent validity (Hair et al., 2019), while FL values in this study ranged from 0.77 to 0.85 were all above 0.50. In addition, Average Variance Extracted (AVE) can be used to determine the convergent validity of the constructs, with AVE values above 0.5 indicating good convergent validity (Hair et al., 2011). As can be seen from Table 3, the AVE values in this study ranged from 0.60 to 0.73, all of which met the criteria suggested by scholars. Again, statisticians suggest that the value of the square root of AVE for a construct should be greater than the value of Pearson’s correlation coefficient for the remaining constructs to indicate good discriminant validity. As can be seen in Table 4, all the constructs in this study showed validity.

**TABLE 4:** Discrimination validity analysis.

Construct		1	2	3	4	5	
1.	Behavioral engagement	(0.77)					
2.	Emotional engagement	0.49***	(0.77)				
3.	Cognitive engagement	0.42***	0.73***	(0.80)			
4.	Job stress	–0.24	–0.39***	–0.37***	(0.83)		
5.	Sustainable well-being	0.31	0.51***	0.54***	–0.55***	(0.85)	
6.	Age	0.34***	0.37***	0.39***	–0.41***	0.45***	(1)

***p < 0.001.

### Model fit analysis

Structural equation modeling (SEM) is commonly used to measure the overall fitness of the study model and model fit is used to confirm the variance acceptance of the study model data (Stanley and Edwards, 2016). The value of χ^2^/*df* must be less than 5 as recommended by statisticians (Hair et al., 2010); RMSEA should be less than 0.1; GFI, AGFI, NFI, NNFI, CFI, IFI, and RFI should all have values greater than 0.800 (Abedi et al., 2015), while PNFI and PGFI should have values greater than 0.500 (Hair et al., 2010). The AMOS 25.0 statistical software was used to conduct the analysis. In the present study, χ^2^/*df* = 3.24, RMSEA = 0.07, GFI = 0.85, AGFI = 0.82, NFI = 0.88, NNFI = 0.90, CFI = 0.91, IFI = 0.91, RFI = 0.87, PNFI = 0.80, and PGFI = 0.72, all of which met the criteria recommended by scholars and had good model fitness.

### Path analysis

**FIGURE 2:**
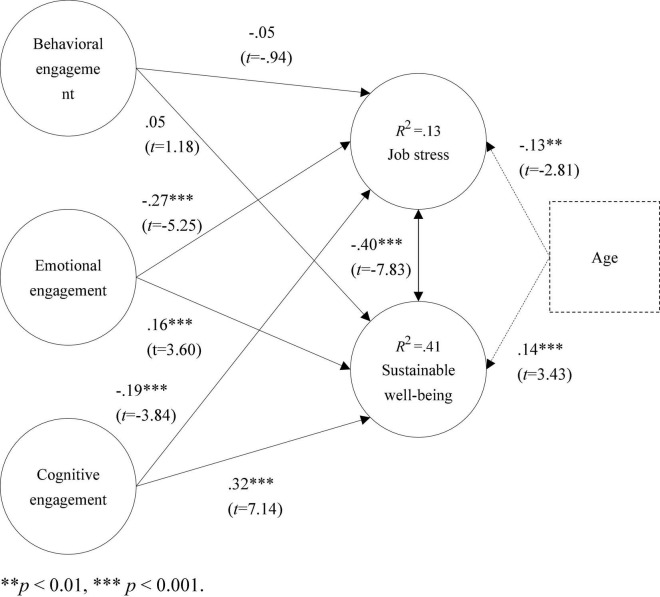
Validation of the research model. ^**^p < 0.01, ^***^p < 0.001.

This study proposes seven research hypotheses based on the engagement theory proposed by Fredricks et al. (2004) and tested by structural equation modeling. Model verification results showed that behavioral engagement did not have a significant effect on job stress (β = −0.05; *t* = −0.94) or on sustainable well-being (β = 0.05; *t* = 1.18). That is, behavioral engagement is not effective in relieving teachers’ work stress and in promoting teachers’ sustainable well-being; Emotional engagement had a negative effect on job stress (β = −0.27^***^; *t* = −5.25), but a positive effect on sustainable well-being (β = 0.16^***^; *t* = 3.60). That is, teachers have a higher level of emotional engagement in leisure activities, which not only helps to reduce their work stress, but also enhances their perceived sustainable well-being; Cognitive engagement had a negative effect on job stress (β = −0.19^***^; *t* = −3.84) but a positive effect on sustainable well-being (β = 0.32^***^; *t* = 7.14). That is, the more cognitively engaged preschool teachers can be in leisure activities, the less stressful their work will be and the higher their level of sustainable well-being will be; and job stress had a negative effect on sustainable well-being (β = −0.40^***^; *t* = −7.83). That is, when the work stress of preschool teachers is relieved, it will help to improve their sustainable well-being. In addition, age had a negative effect on job stress (β = −0.13^**^; *t* = −2.81) but a positive effect with sustainable well-being (β = 0.14^***^; *t* = 3.43), as shown in Figure 2.

In addition, when the explanatory power values are in the range of 0.25,0.50, and 0.75, they represent weak, medium and strong levels of explanatory power respectively (Hair et al., 2010). In this study, the explanatory power of job stress was 13% and the explanatory power of sustainable well-being was 41%, indicating that this study found a weak and moderate degree of explanatory power, as shown in Figure 2.

### Discussion

Based on the engagement theory proposed by Fredricks et al. (2004), this study conducted a questionnaire survey among preschool teachers over the age of 18 to explore the relationship between three types of leisure engagements, including behavioral, emotional, and cognitive, and preschool teachers job stress and sustainable well-being. Ensuring healthy lifestyles is particularly important for people’s sustainable well-being in light of SDG 3 for 2030 (Sweileh, 2020). While different age groups have different leisure activity participation (Kim et al., 2018), more importantly, leisure activity engagement and well-being levels change with age (Cocozza et al., 2020). In addition, different age groups such as young, middle-aged, and older people tend to have different levels of leisure engagement that continue to contribute to their physical and mental health and well-being status, which is consistent with previous research (Chen et al., 2020). That is, teachers of young children at different ages will have different levels of leisure engagement and sustainable well-being. Therefore, this study used age as a controlled variable, and after excluding the effect of age, this study yielded more accurate results.

#### Preschool teachers’ leisure engagement is not related with job stress and sustainable well-being

According to Kim and Brown (2018), leisure activities are seen as an important factor in health and engaging in more leisure activities would be beneficial for reducing mental health problems such as stress and anxiety. In addition, Nagata et al. (2020) argued that leisure engagement, as an important part of leisure activities, are the frequency and behavioral expression of people’s participation in leisure activities. However, the results of this study showed that leisure engagement was not related with preschool teachers’ job stress or sustainable well-being. This shows that not all types of leisure engagement are related to their work stress and psychological state. For example, Schnitzler et al. (2021) argued that engagement is a complex and variable construct, and that behavioral, emotional, and cognitive engagement can inconsistently affect behavior and outcomes. The three levels of engagement differ, people’s behavioral engagement is more superficial, whereas emotional and cognitive engagement influence their engagement effects in a more profound way (Liu et al., 2022). In addition, Matsumoto et al. (2022) argued that behavioral engagement in leisure activities may not completely release stress and also this behaviorally oriented behavioral engagement in leisure does not adequately explain well-being.

Furthermore, due to the impact of the COVID-19 pandemic, outdoor, physical, recreational, and social activities have been limited (Liu et al., 2021), preventing many preschool teachers from engaging in timely leisure activities and thus limiting the frequency and opportunities for behavioral engagement, which may be a significant factor in their inability to effectively relieve work stress and maintain a sustained sense of well-being (Randall et al., 2021). This may be a significant factor in their inability to effectively reduce work stress and maintain sustainable well-being (Randall et al., 2021). Moreover, Petrić et al. (2022) noted that if preschool teachers are only involved in leisure activities and do not really enjoy or recognize their importance, they will not be able to generate lasting motivation to engage in leisure activities and also to relieve the tensions associated with their job stress. Those preschool teachers who enjoy participating in leisure activities tend to believe that emotional engagement in leisure activities is more important than behavioral engagement and that participation in leisure activities such as hiking and travel can provide a sense of pleasure and satisfaction. In addition, those who value leisure activities also consider them as ordinary exercise activities, such as participation in sports, recreation and relaxation, which can promote relaxation and continue to influence their physical and mental health (Matsumoto et al., 2022). All of the above illustrate that leisure behavioral engagement is not necessarily important for preschool teachers and that emotional and cognitive engagement are more conducive to soothing their minds and bodies so that they continue to have pleasant, positive emotional experiences. Therefore, the results of this study do not support research hypotheses 1 and 2. That is, leisure behavioral engagement was not related with the job stress and sustainable well-being of preschool teachers.

#### Preschool teachers’ emotional engagement is negatively related to job stress

According to Duan and Mu (2018), individuals are often prone to high levels of stress when their resources differ from their needs. Chen et al. (2020) also pointed out that engaging in leisure activities is enjoyable and more emotional involvement in leisure activities may help people to relieve their mind and body. For example, those who have higher emotional involvement in leisure activities are more likely to engage in leisure activities such as recreation and sports to reduce negative effects such as work stress. In addition, Hong et al. (2022) also pointed out that preschool teachers, as the main bearers of responsibility for preschool children’s care and education, are often in a state of stress and overload due to the special nature of their educational targets and emotional factors are not only important predictors of their psychological health, but also alleviate their stress and tension to some extent. Moreover, Gu et al. (2020) indicated that for preschool teachers in a high stress state, kindergarten administrators should provide more emotional support and offer more time and space for leisure and relaxation, thus helping them to recover from their negative work stress state. Therefore, the present study is consistent with the previous studies and supports research hypothesis 3. That is, more emotional engagement of preschool teachers in leisure activities would help reduce their work stress.

#### Preschool teachers’ emotional engagement is positively related with sustainable well-being

According to Wang et al. (2020), well-being is the ability to maintain optimal psychological functioning and positive emotional experiences when people experience challenges and change. In addition, Sheldon and Lyubomirsky (2021) stated that sustainable well-being is critical to people’s health and future development, emphasizing the ability to continue to have a stable, positive emotional experience in their future work lives. Furthermore, Eckhaus and Sheaffer (2019) argued that emotional factors are important factors influencing sustainable well-being because having positive emotional experiences motivates people to work, have sustained motivation and thus meet future challenges and difficulties in a better state. For example, those preschool teachers who enjoy participating in leisure activities may expand their opportunities for social interaction through leisure activities and leisure communication, engage in more leisure activities to soothe their minds and bodies and more quickly eliminate the effects of negative life events to maintain sustainable well-being. Therefore, this study is consistent with the previous studies and supports hypothesis 4. That is, more emotional engagement of preschool teachers in leisure activities will contribute to their sustainable well-being.

#### Preschool teachers’ cognitive engagement is negatively related to job stress

Wu et al. (2021) suggested that the perception of leisure activities may influence people’s physical and mental health. In particular, when people believe that leisure activities such as social and recreational activities can relieve their mental and physical stress to a certain extent, it can satisfy their psychological needs when they are faced with heavy workloads and demands (Ihle et al., 2020). Furthermore, Poulin et al. (2019) indicated that supporters who value leisure activities invest more time and energy in them and participating in leisure activities will reduce the negative emotional impact of their work. Therefore, Rodríguez-Rey et al. (2020) suggested that leisure cognitive engagement would facilitate the adjustment of negative emotions and have an important impact on reducing work stress. Petrić et al. (2022) stated that preschool teachers tend to neglect the important value of leisure activities due to the specificity of their work and if they can draw more attention to leisure activities, by participating in recreational, sports, social and other leisure activities, they may further relieve their high level of work stress and satisfy their psychological needs. Therefore, the present study is consistent and supports hypothesis 5. That is, more cognitive engagement of preschool teachers in leisure activities would be beneficial for reducing their work stress.

#### Preschool teachers’ cognitive engagement is positively related with sustainable well-being

As seen in the agenda of SDG 3, healthy lifestyles are particularly important for people’s sustainable well-being (Sweileh, 2020). This is because people can relax from leisure activities and continue to be energetic and enthusiastic in their future work life by participating in healthy habits such as leisure activities (Sala et al., 2019). Thus, in addition, Bailey et al. (2016) stated that leisure cognitive engagement is crucial for people’s well-being. Since people who value leisure activities will better understand the relationship between leisure activities and their health, they will continuously maintain positive emotional experiences through leisure activities such as social interaction. Therefore, Petrić et al. (2022) suggested that only by preschool teachers maintaining a healthy lifestyle, focusing their attention on leisure activities and engaging in more leisure activities to keep both body and mind active, can they be more permanently motivated to cope with complex environments and challenges, thus maintaining a stable, satisfying and positive sense of sustainable well-being. Therefore, the present study is consistent with the previous studies and supports research hypothesis 6. That is, more cognitive engagement of preschool teachers in leisure activities would be beneficial for increasing their sustained well-being.

#### Preschool teachers’ job stress is negatively related with sustainable well-being

Sheldon and Lyubomirsky (2021) proposed that sustainable well-being is an emotional experience that remains stable and positive throughout working life based on the theory of the sustainable well-being model. In particular, preschool teachers often face overload, severe lack of leisure time, and low pay, which may be detrimental to their well-being acquisition (Li and Zhang, 2019). In addition, Hong et al. (2021) indicated that excessive job stress may cause tension in preschool teachers and being in a state of chronic tension and anxiety may affect their sustainable well-being. Furthermore, Tandler et al. (2020) also concluded that work stress was considered a significant predictor of hindrance to well-being. If preschool teachers were given more support, it would be beneficial to help them cope with a variety of challenges and difficulties and further reduce their job stress (Jeon et al., 2018). Therefore, when preschool teachers have lower levels of job stress, they will continue to have a satisfying and positive sense of sustainable well-being. The results of this study thus indicate that job stress is negatively related to the sustainable well-being of preschool teachers, supporting research hypothesis 7. That is, lower job stress among preschool teachers in leisure activities would be beneficial for increasing their sustainable well-being.

## Conclusion and recommendations

### Conclusion

In the context of the SDG 3 agenda, ensuring healthy lifestyles for people is particularly important for the mental health and well-being of educators. Leisure engagement, as a healthy lifestyle, plays an important role in promoting educators’ continued enthusiasm for their work as well as their sustainable well-being. Therefore, this study explores the relationship between leisure engagement and the sustainable well-being of preschool teachers, proposing a research model with seven research hypotheses. The findings showed that (a) behavioral engagement was not related with the job stress or the sustainable well-being of preschool teachers; (b) emotional engagement was negatively related with the job stress but positively related with the sustainable well-being of preschool teachers; (c) cognitive engagement was negatively related with job stress of preschool teachers, but positively related with sustainable well-being; and (d) job stress was negatively related with the sustainable well-being of preschool teachers.

From the results of the study, not all engagement can bring positive results. Behavioral engagement is an initial engagement, so its effect is quite limited, while emotional and cognitive engagement is a higher level engagement type, which also brings a greater effect. It is clear that emotional and cognitive engagement are more conducive to reducing the job stress of preschool teachers, as well as promoting a sense of sustainable well-being, which leads to sustainable passion, pleasure and well-being in their current and future lives. In addition, this study uses different types of leisure engagement to understand the relationship with job stress and well-being, which helps to enhance the effectiveness of preschool teachers when they participate in leisure activities.

### Recommendations

Past research has often explored the relationship between stress and people’s mental health in work situations, as well as assessing their work stress status. However, with the introduction of the 2030 SDG 3 agenda, the focus on healthy lifestyles and people’s well-being has become increasingly popular and highly discussed (Sweileh, 2020). Therefore, exploring the relationship between leisure engagement and the sustainable well-being of preschool teachers will help expand the application of healthy lifestyles in the field of education. Moreover, because of the special nature of their work, teachers are often under high levels of stress and tension, and by engaging in leisure activities, not only does it provide a richer resource for preschool teachers, but it also helps preschool teachers balance their work stress and become better prepared to engage in kindergarten education and teaching, thus supporting and promoting preschool teachers’ learning and development. Therefore, it is recommended that kindergarten educators and administrators should value the importance of leisure activities, engage in more leisure activities to soothe their bodies and minds, further relieve their job stress and continue to maintain positive and sustainable well-being.

In addition, Fredricks et al. (2004) proposed engagement theory, which explains the three types of engagement and how they influence people’s behavior and outcomes through them. However, this study found that not all types of leisure engagement relieved the job stress of preschool teachers and that emotional and cognitive engagement were more conducive to their sustainable well-being in dealing with complex environments and challenges. Therefore, preschool educators and administrators should engage in leisure activities, value the important influence of leisure activities, plan their work, life and leisure time rationally and engage in leisure activities with more enthusiasm to balance job stress and burnout so that they can better continue to maintain a stable and positive sense of sustainable well-being in their future work and life.

### Limitations and future study

Since the data collection period for this study was from January 15 to February 28, 2022, the impact of the COVID-19 pandemic is not yet over and there will be small outbreaks in some countries and regions, among many other conditions. In addition, COVID-19 has become an important factor affecting sustainable well-being in the process of achieving SDG 3 by 2030. However, in the post-epidemic era of repeated COVID-19 outbreaks and localized epidemic prevention, how to maintain a healthy lifestyle and further reduce people’s work stress and tension by engaging in more leisure engagement is also an important issue worth exploring in subsequent studies.

According to the engagement theory proposed by Fredricks et al. (2004), people’s engagement states may be influenced by different factors which affect individual behaviors and outcomes through positive interactions with individual environments and systems (Wang and Fredricks, 2014). In addition, organizational resources have been found to favorably enhance people’s engagement states, further satisfying their work demands and engaging in their work with more enthusiasm and energy (Lacanienta et al., 2018). Therefore, the relationship between organizational resources for leisure engagement and sustainable well-being may be explored in subsequent studies.

Moè and Katz (2020) and Moè and Katz (2021) showed that emotion regulation as a form of adaptive emotion regulation has an important role in teachers’ need satisfaction, motivational teaching style and work status. In different periods of time, organizations may use emotion regulation to increase job satisfaction and engage in work with more positive emotions, thus further reducing the risk of turnover intentions (Cote and Morgan, 2002). Related research also suggests that emotion regulation can further contribute to well-being by meeting basic psychological needs (Benita et al., 2020), but in this study, the emotion regulation variable was not included. Therefore, emotion regulation can be used as a moderating variable in the follow-up study to explore the impact on the relationship between leisure engagement and sustained well-being of preschool teachers.

In addition, questionnaires are often used to measure and validate people’s behaviors, attitudes and characteristics, and are unable to better interpret phenomena and the correlations underlying them, or to explore in depth the different relationships that different types of leisure engagement have on the sustainable well-being of preschool teachers. Therefore, in-depth interviews may be used in future studies to understand preschool teachers’ perceptions of the three types of leisure activity engagement and sustainable well-being in order to extend the results of this study.

## Data availability statement

The raw data supporting the conclusions of this article will be made available by the authors, without undue reservation.

## Ethics statement

Ethical review and approval was not required for this study on human participants in accordance with the local legislation and institutional requirements. Written informed consent for participation was not required for this study in accordance with the national legislation and the institutional requirements.

## Author contributions

All authors listed have made a substantial, direct, and intellectual contribution to the work, and approved it for publication.

## Conflict of interest

The authors declare that the research was conducted in the absence of any commercial or financial relationships that could be construed as a potential conflict of interest.

## Publisher’s note

All claims expressed in this article are solely those of the authors and do not necessarily represent those of their affiliated organizations, or those of the publisher, the editors and the reviewers. Any product that may be evaluated in this article, or claim that may be made by its manufacturer, is not guaranteed or endorsed by the publisher.
